# Laminate veneer ceramics in aesthetic rehabilitation of teeth with fluorosis: a 10-year follow-up study

**DOI:** 10.1186/s12903-022-02079-4

**Published:** 2022-02-17

**Authors:** Zeynep Basagaoglu Demirekin, Suha Turkaslan

**Affiliations:** Department of Prosthodontics, Suleyman Demirel University Faculty of Dentistry, Isparta, Turkey

**Keywords:** Fluorosis, Laminate veneer ceramics, Aesthetic rehabilitation

## Abstract

**Background:**

Fluorosis is one of the color anomalies seen in teeth. White lines and blurred areas associated with mild fluorosis are barely noticeable; in its severe form, tooth enamel surface changes ranging from staining and pitting may be observed. The treatment of fluorosis not only provides aesthetic and functional correction but also helps to improve the patient's self-esteem.

**Methods:**

The present retrospective study evaluated the clinical quality, success rate, and estimated survival of porcelain laminate veneers in teeth with anterior fluorosis. Three hundred fifty-eight porcelain laminate veneers (254 in the anterior maxilla and 104 in the mandible) were "functional" restorations that covered the incisal edge and part of the palatal/lingual side of the tooth with a 1 mm high palatal bevel. Ceramic veneers were fabricated with lithium disilicate reinforced glass–ceramic material (IPS e.max Press, Ivoclar Vivadent, Schaan, Liechtenstein). The modified United States Public Health Service criteria were used for clinical evaluation of the restorations.

**Results:**

On the basis of these criteria, marginal adaptation, color match, marginal discoloration, surface roughness, restoration fracture, tooth fracture, restoration wear, antagonist tooth wear, caries and postoperative sensitivity were evaluated yearly. The survival rate in the current study was ≥ 0.997 for 10 years.

**Conclusions:**

The results of this clinical study should encourage clinicians to consider ceramic veneers over crown restorations when restoring the smile of patients with advanced fluorosis.

## Background

Smiling is known to benefit general human health by increasing happiness along with the secretion of endorphins, reducing stress and blood pressure [1]. Smiling relies on essential components such as the color and proportion of the teeth, structure of the gingival tissue, and the lip frame [2]. An unpleasant smile can affect the patient's self-esteem. A frequently encountered complaint is the change of tooth color, which is one of the most critical components of the ideal smile. Fluorosis is one of the color anomalies seen in teeth. It is a developmental disorder of the enamel that affects ameloblasts at the mineralization stage due to systemic fluoride intake that is greater than the optimal of 1 ppm/day [3, 4].

The outer layer of enamel tissue that is exposed to the bacterial flora is characterized as hypermineralized and acid-resistant. On the other hand, the subsurface layer is more porous and has homogeneously distributed hypomineralization areas [5, 6]. White lines and blurred areas associated with mild fluorosis are barely noticeable; in its severe form, tooth enamel surface changes ranging from staining and pitting may be observed [5, 7, 8]. The treatment of fluorosis not only provides aesthetic and functional correction but also helps to improve the patient's self-esteem [9, 10].

In cases of generalized dental fluorosis with surface defects, aesthetic, phonetic, and functional rehabilitation requires the removal of unsupported and pitted enamel or dentin tissue (with or without the occlusal vertical dimension) and fabrication of crowns or ceramic veneers [11]. Porcelain laminate veneers have been a viable option for restoring anterior teeth for almost three decades due to their aesthetic appeal, durability, and biocompatibility. Today, ceramic veneers are mainly used to optimize tooth form and position, close diastemas, replace unaesthetic composite resin restorations, restore teeth with incisor wear or tooth erosion and mask or reduce tooth discoloration [12, 13]. They are a valid alternative to full-covered restorations, as they avoid aggressive tooth preparations and thus preserve the tooth structure [14, 15].

Dental fluorosis can be mild or severe, depending on various factors, including duration and amount of fluoride exposure, individual differences, weight, age, degree of physical activity, nutritional factors, and bone growth [16]. The mild form of fluorosis may appear as small, unnoticeable white streaks. In moderate-to-severe fluorosis, brownish discoloration can be seen with pitting and abrasion on the enamel surface due to poor mineralization of the enamel [17, 18]. Therapeutic management of dental fluorosis depends on the severity of the condition. In severe cases, the use of the Thylstrup and Fejerskov Index (TFI), which characterizes changes in the enamel surface, should be considered while determining the type of treatment. The severity of fluorosis was classified with the Thylstrup and Fejerskov index (TFI) based on biological changes in the tooth enamel surface. The severity was considered to be mild when TFI = 1–3, moderate with TFI = 4–5 and severe with TFI > 6 [19]. The present retrospective study evaluated the clinical quality, success rate, and estimated survival of porcelain laminate veneers in teeth with anterior fluorosis. The study's null hypothesis is that in the long term, such as ten years, anterior porcelain ceramic laminates on fluorized teeth will show low success and survival rate.

## Methods

### Inclusion criteria of patients

Inclusion criteria were as follows: all subjects were at least 18 years of age, able to read and sign the informed consent form, physically and psychologically able to tolerate conventional restorative procedures, free of active periodontal or pulpal disease, with existing resin restorations in good condition, and were willing to undergo follow-up examinations as indicated by investigators. Patients with a history of parafunctional habits were excluded from the study. Each patient was also informed of alternative treatment options.

Existing composite restorations, visible caries, pitting, or marginal staining were removed prior to the preparation of the tooth.

### Clinical examination

Thirty-four patients (20 women, 14 men; mean age 49.7 years, range: 19–60 years) who were treated in our clinic between March 2011 and December 2011, needed ceramic veneer restorations and met the inclusion criteria, were included in this study. All patients were given an informed consent form approved by the institutional review ethics committee of our university (ABR number: 72867572-050.01.04-55786). For all of the teeth included in the current study, the TFI was moderate (TFI: 4–5).

Three hundred fifty-eight porcelain laminate veneers (254 in the anterior maxilla and 104 in the mandible) were "functional" restorations that covered the incisal edge and part of the palatal/lingual side of the tooth with a 1 mm high palatal bevel. Among the anterior maxillary restorations, 68 were carried out on the central incisors, 68 on the lateral incisors, 58 on the canines, and 60 on the premolars. In the mandible, 26 crowns were located on the central incisors, 26 on the lateral incisors, 26 on the canines, and 26 on the premolars (Table 1).

**Table 1:** The restorations carried out relative to the location of the teeth

	Central incisors	Lateral incisors	Canine	Premolar	Total number of ceramic veneers
Maxillary arch	68	68	58	60	254
Mandibular arch	26	26	26	26	104
Total number of ceramic veneers	94	94	84	88	358

### Pre-treatment procedures

Prior to treatment with ceramic veneers, gingival corrections, teeth whitening procedures, and orthodontic alignment arrangements were carried out as necessary. Aesthetic evaluations were carried out on plaster models and digital photographs taken in the articulator using face-bow recordings. The color was determined under standard conditions in the dental laboratory. Wax modeling was carried out on the plaster model using the mock-up technique and was necessary to communicate details on correcting the form and position of the teeth with the patient and the technician and evaluating the patient's expectations. After obtaining approval of the mock-up from the patient, dental preparations were initiated.

### Tooth preparation

**Fig. 1:**
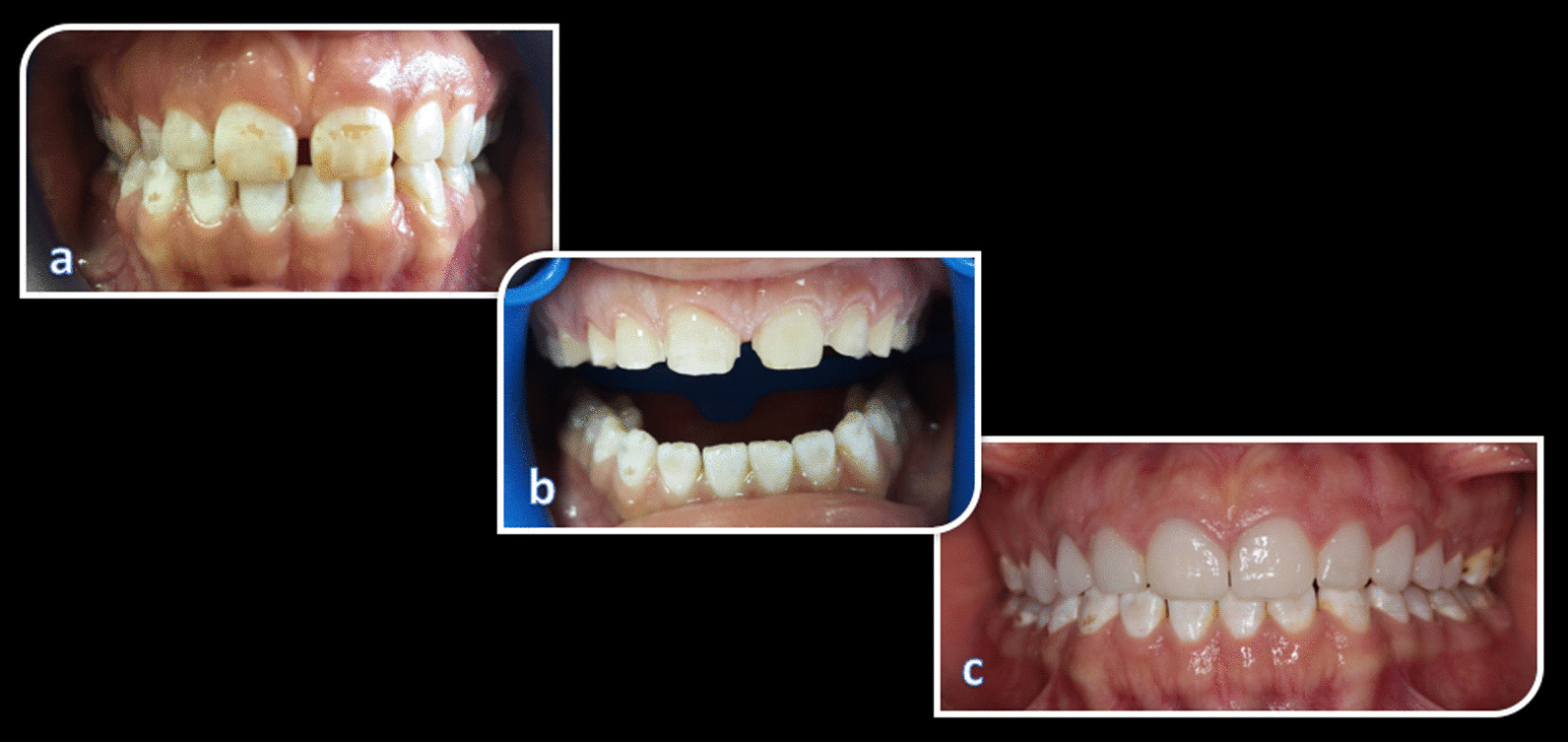
**a** before, **b** preperation, **c** after

Surface preparation for the rehabilitation of teeth with moderate fluorosis using ceramic veneers are well established general procedures. In this context, the cut was kept minimal, and restricted to the enamel surface as much as possible. It has been shown in the literature that window-type labial enamel preparation should be avoided; incisal bevel preparation is preferred instead. To prepare the tooth, three horizontal surface depths of 0.3 mm at the enamel-cementum junction, 0.5 mm at the middle third, and 0.7 mm at the incisal third were used for a depth-limiting bur. Depth grooves were marked with a pencil. A retraction cord (Ultrapak Cord #00, Ultradent Products Inc., South Jordan, UT, USA) was placed for protecting the gingival tissue. The cutting edge was reduced by 1.5 mm with a diamond bur to create a distinctive chamfer finish line continuous with the facial-proximal outline. The preparation was more extensive due to brown staining on the mesial and distal proximal surfaces while the cervical margin was at the gingival level (Fig. 1). After the incisal edge angles were rounded, the exposed dentin areas were determined, and immediately closed with a universal adhesive. Following this, the final preparation and occlusion were evaluated.

### Final impressions and temporary restorations

Impressions were taken with a polyvinylsiloxane material (Virtual, Ivoclar Vivadent, Amherst, NY) according to the manufacturers instructions. The labial surface of each prepared tooth was spot etched with 37% phosphoric acid (Self Etch, Kuraray Dental, Japan) and a bonding agent (Clearfil SE Bond, Kuraray Dental, Japan) was applied to these spots. A high-intensity light-emitting diode (LED) light was applied for 20 s (Elipar S10, 3 M ESPE, MN, USA). A prefabricated transparent matrix (3 M ESPE, MN, USA) was loaded with a temporary resin material and placed on the tooth. Light curing was applied for 10 s to each tooth.

### Adhesive cementation

Ceramic veneers were fabricated with lithium disilicate reinforced glass–ceramic material (IPS e.max Press, Ivoclar Vivadent, Schaan, Liechtenstein). Temporary veneers were removed, and the teeth were cleaned using polishing paste and prophylaxis brushes. A clear trial resin (Variolink Veneer try-in paste, Ivoclar Vivadent, Schaan, Liechtenstein) was used to assess marginal adaptation and color of the veneers. Next, the inner surface of the restoration was prepared for cementation. After etching with hydrofluoric acid (Ceramic Etchant 9.5%, Bisco Inc., Schaumburg, IL, USA) for 60 s, the area was washed with pressurized water for the same duration as the acid etching and dried with an air–water spray. Silane (Clearfil SE Bond KIT, Kuraray Dental, Japan) was applied for 60 s to the inner surface that would come in contact with the tooth, followed by a short duration of air drying. A layer of bonding agent (Clearfil SE Bond KIT, Kuraray Dental, Japan) was applied to the prepared tooth surfaces and thinned with air. Following this, Heliobond (Ivoclar Vivadent, Schaan, Liechtenstein) was placed on the prepared tooth surfaces. The inner surface was coated with light-cured resin cement (Variolink Veneer, transparent shade, Ivoclar Vivadent, Schaan, Liechtenstein). Veneers were appropriately placed on the tooth by applying gentle pressure. Next, light was applied on each laminate veneer from the facial and lingual regions for 40 s.

### Evaluation

**Fig. 2:**
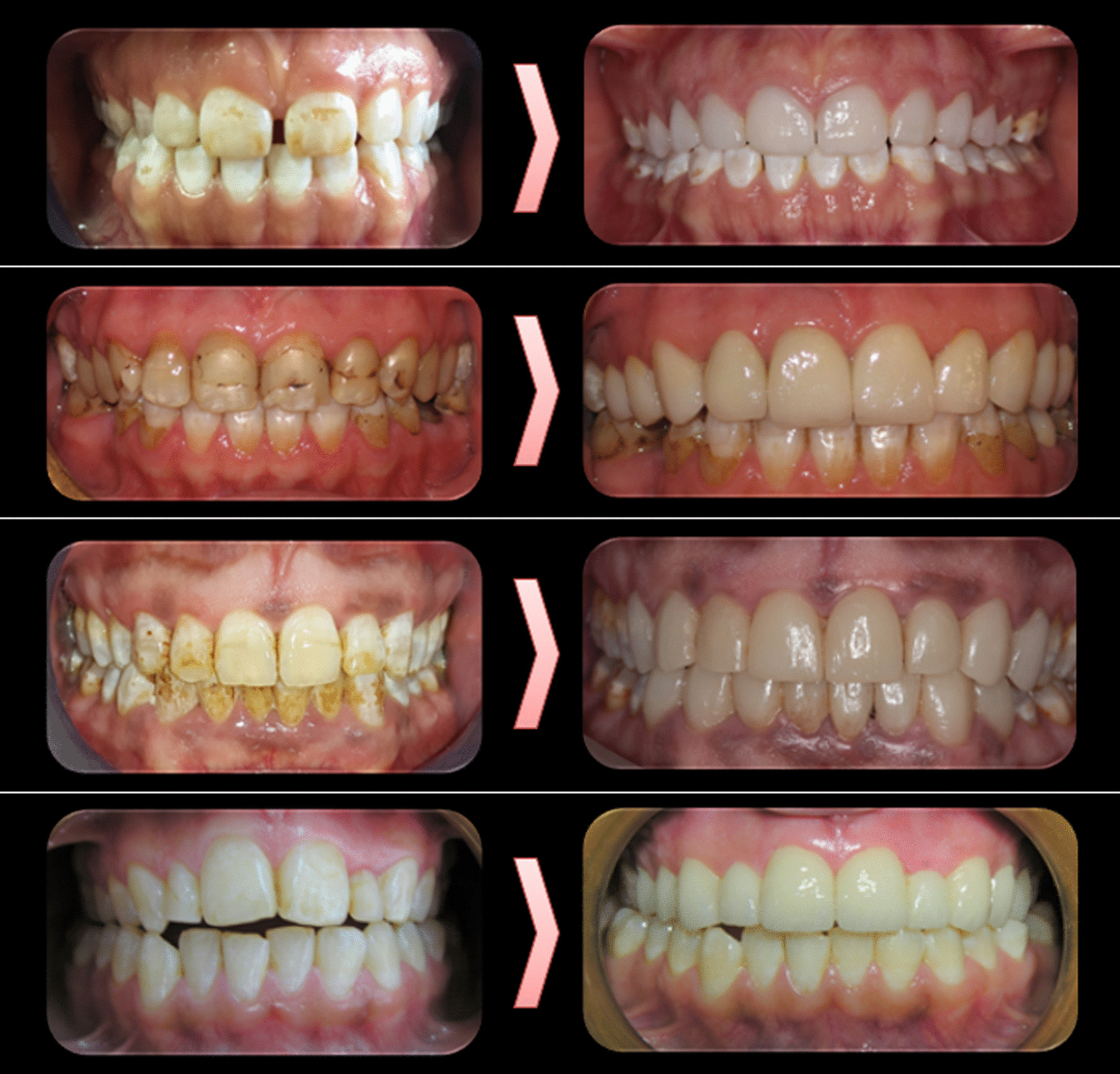
Before–after photos in 10-year follow-up results

The modified United States Public Health Service criteria were used for clinical evaluation of the restorations. On the basis of these criteria, marginal adaptation, color match, marginal discoloration, surface roughness, restoration fracture, tooth fracture, restoration wear, antagonist tooth wear, caries and postoperative sensitivity were evaluated yearly (Fig. 2).

### Statistical analyses

A descriptive approach was adopted for statistical analysis. Descriptive statistics are presented as numbers and percentages. A sample size of 3960 achieves 100% power to detect an effect size (W) of 0.1129 using a 19 degrees of freedom Chi-Square Test with a significance level (alpha) of 0.05000. The Kaplan Meier method was used to analyze the survival rates of ceramic laminates. Survival time was defined as the time from cementation to failure of the restoration. The main criterion used to define the failure of the veneer was fracture of the ceramic and/or deterioration of adhesion [20].

## Results

The findings observed during the follow-up period for the teeth numbered 45, 35, 15, 14 are shown in Table 2.

**Fig. 3:**
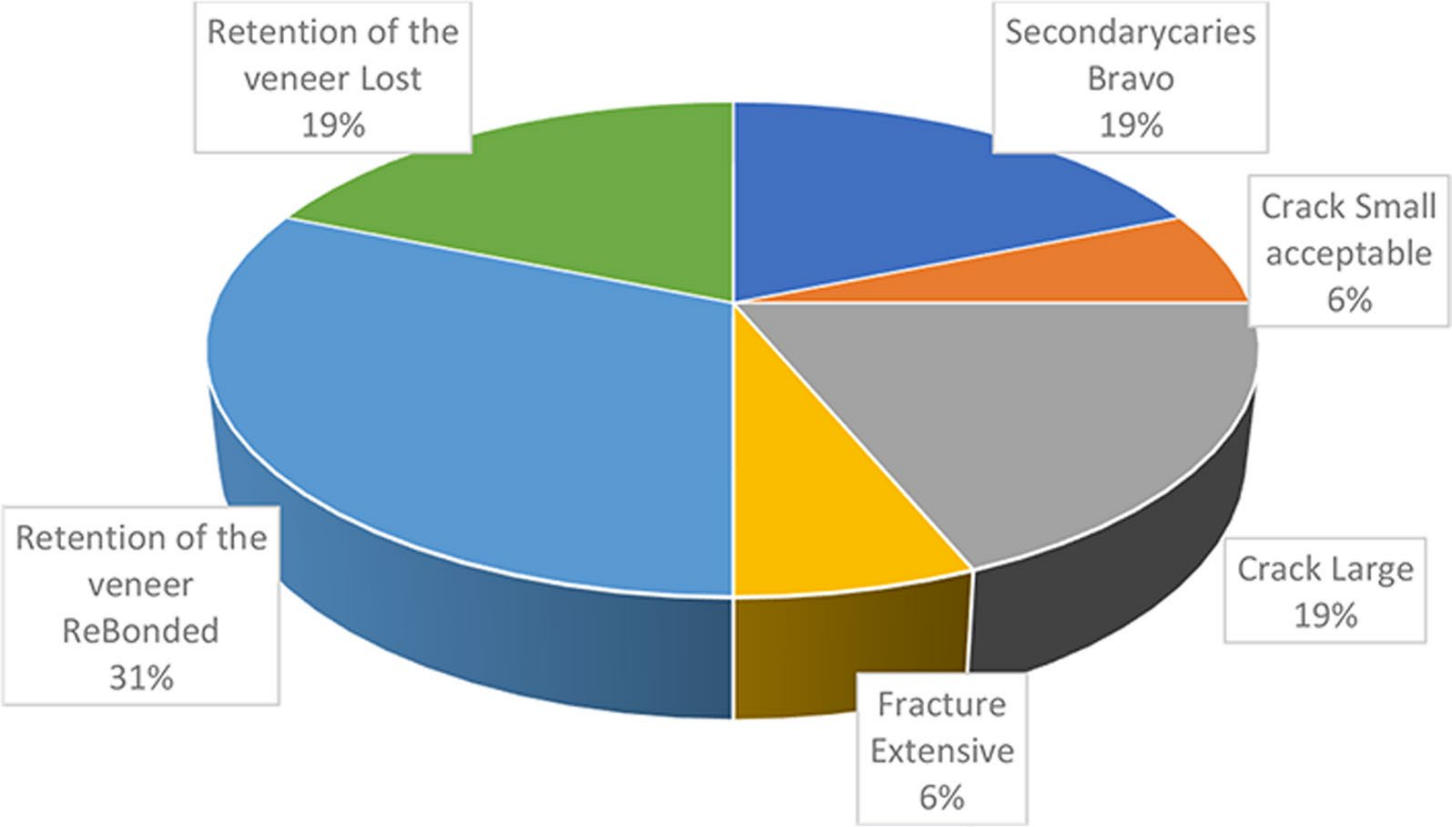
Distrubution of the different failures observed with the laminate veneers

- According to Table 2, the survival rate in the current study was ≥ 0.997.
- Retention of the veneer ReBonded was observed in teeth 34 and 45 at the 12th month follow-up, while small acceptable fractures were detected in tooth 35 at the 24th-month follow-up.
- At the 48th month follow-up, the veneer ReBonded and retention of the secondary caries Bravo was observed in teeth 15 and 14.
- At the 84th month follow-up, secondary caries Bravo, large cracks, Retention of the veneer Lost were detected in tooth 35.
- At the 96th month follow-up, large cracks, widespread fractures and Retention of the veneer Lost were detected in tooth 45.
- At the 108th month follow-up, retention of the veneer Lost, and large cracks were detected in tooth 45.
- Distribution of the different failures observed with the veneers (Fig. 3)

**Table 2:** Descriptive statistics and estimated survival rate for the variables studied

	Number of healthy teeth	Number of falling teeth	Tooth number	Time (months)	Estimate of cumulative proportion of survival
Secondary caries Bravo	3957 (99.92)	3 (0.08)	15	48	0.999
			14	48	
			35	84	0.999
Crack Small acceptable	3959 (99.97)	1 (0.03)	35	24	1.000
Crack Large	3957 (99.92)	3 (0.08)	35	84	0.999
			45	96	0.998
			45	108	0.997
Fracture Extensive	3959 (99.97)	1 (0.03)	45	96	0.999
Retention of the veneer ReBonded	3955 (99.87)	5 (0.13)	34	12	0.999
			45	12	
			45	12	
			15	48	0.998
			14	48	
Retention of the veneer lost	3957 (99.92)	3 (0.08)	35	84	0.999
			45	96	0.998
			45	108	0.997

## Discussion

Dental fluorosis is known as a developmental disorder of the enamel. Clinical studies evaluating the effectiveness of available techniques for the treatment of fluorosis are lacking in the literature, and long-term results have not been demonstrated. Most published studies are clinical case reports, making it difficult to compare the findings of the current study with those in the literature [21–24]. The current study aimed to evaluate the long-term outcome of treatment approaches with ceramic veneers in patients with dental fluorosis. Based on the findings of this clinical trial, our null hypothesis was rejected.

Dental fluorosis affects primarily those individuals who live in areas with excessive fluoride in their drinking water. It is defined as developmental disorder of the tooth enamel due to excessive fluoride exposure, and is usually manifested in pediatric patients [25]. During tooth development, a high fluoride concentration can particularly affect cells called ameloblasts that are responsible for enamel formation [26]. Interactions between the mineral matrix of the developing enamel and ameloblasts lead to the changes observed in the enamel. Increased fluoride intake during enamel mineralization is accompanied by a decrease in free calcium ion concentration in the mineralizing matrix, which prevents enzyme proteases from breaking down matrix proteins during the maturation phase [27–30]. Therefore, the degradation of matrix proteins is delayed [27]. In addition, the presence of fluoride-induced retention of enamel matrix proteins such as amelogenins, ameloblastins, tuftelins, enamelin, and high molecular weight sulfated proteins leads to impaired crystal growth [18, 27].

The classification criteria established by Thylstrup and Fejerskov can be used to determine treatment modalities based on the biological aspects of dental fluorosis [10]. Bleaching and enamel microabrasion are non-invasive procedures that are recommended for cases with mild to moderate fluorosis with a TFI score of 0–4 [31]. Based on recommendations by Akpata, mild fluorosis with a TFI score of 1–2 can be treated with bleaching [32]. Bleaching agents can ameliorate external stains in cases where the porosity is superficial [33]. Subsurface porosity is known to be deep in cases with TFI scores of 3–4; in such cases, bleaching alone is unlikely to be effective. Thus, a combination of bleaching with microabrasion can be used in such cases to remove fluorosis stains. The porous enamel region is located approximately 80-100 μm below the surface enamel in teeth with a TFI score of 1–3. Since micro-abrasion causes an approximately 100 μm loss of surface enamel it should be recommended for the treatment of mild fluorosis. Teeth with a TFI score of 5–7 should be treated with ceramic veneers, while teeth with a TFI score of 8–9 should be crowned because more than 50% of the enamel is lost [32, 33].

Treatment options for dental fluorosis include micro/macro etching, bleaching, composite restorations, veneers, and full crowns. Other conservative techniques for treating dental fluorosis include composite veneers, resin infiltration, and tooth jewelry. Since dental fluorosis is an endemic public health problem, availability of such treatment options can provide better outcomes in patients [10, 34]. However, no randomized controlled trials have been conducted to date to evaluate treatment approaches such as crowns and ceramic veneers. Based on the classification criteria of Thylstrup and Fejerskov, ceramic veneers were chosen as the best treatment option to mask tooth discoloration in dental fluorosis patients in the current study [10, 35]. As with any technique in development, the use of ceramic coatings requires both medium-term and long-term studies to confirm their usefulness and ensure their continued use [20]. The treatment protocols used in the current study were aimed to improve the smile of the patient and provide aesthetic rehabilitation of the teeth. This aim was achieved by using ceramic veneers, which is the treatment of choice to mask tooth discoloration in cases with moderate fluorosis. Because ceramic veneers require a minimally invasive design, they can completely mask the discolored tooth with minimal reduction of intact tooth material, providing predictable and long-term aesthetic rehabilitation. Glass–ceramics have excellent translucency; moreover, the presence of 35% leucite crystals can reflect light and contribute to the durability of the material. The bonding mechanism of enamel is more effective compared to dentin [11]. Correct choice of ceramic material can allow stable aesthetic qualities whilst maintaining biocompatibility, abrasion resistance, good translucency, color, and contour stability. In addition, the risk of gingival irritation may be reduced due to lower plaque deposition around ceramic veneers compared to natural teeth [36, 37].

Magne and Douglas reported that the average local flexibility of teeth was doubled with the removal of the outer enamel, and tooth hardness was fully restored after ceramic veneer bonding. These data strongly suggest that restorations can mimic the biomechanical properties and structural integrity of the original tooth and confrm their biomimetic behavior [36, 38].

Ceramic veneers are primarily used for aesthetic reasons. Therefore, they can be considered to be elective rather than necessary for the maintenance of health, at least in terms of removing and restoring diseased tooth tissue. However, despite the more minimally invasive nature of its preparation when compared to a crown, the veneered tooth very often requires restorative dentistry [39]. For these reasons, the longevity of a ceramic veneer is of utmost importance. The durability and clinical success of ceramic coatings have been extensively studied in the literature. Ceramics veneers have been reported to provide durable and successful restoration with an estimated survival probability of 93.5% over ten years. Satisfactory results were obtained over a six-year follow-up period for restoration of teeth with fluorosis with ceramic crowns. In addition, numerous studies have shown acceptable aesthetic results with restorations using ceramic veneers in cases with moderate to severe fluorosis [34].

Improvements in clinical performance must address the root causes of failure. The leading causes of failure observed in clinical trials are still secondary caries in the tooth and fractures in the restoration. In the current study, the teeth with the highest rate of failure of restoration observed with long-term follow-up were the mandibular premolars. Thus, mandibular premolars are less resistant to occlusal loads than other teeth due to their narrower counter-occlusal layer. In addition, the widespread restoration failure in the mandibular premolar region may also have emanated from the difficulties in maintaining proper oral hygiene in this region.

## Conclusions

Ceramic veneers are considered to be one of the most popular restorative materials in aesthetic dentistry. They provide excellent outcomes when an appropriate treatment plan and protocol are used during the clinical and laboratory production stages. The current study recommends the use of ceramic veneers to improve the appearance of fluorotic teeth, thereby improving the patient's smile. The results of this clinical study should encourage clinicians to consider ceramic veneers over crown restorations when restoring the smile of patients with advanced fluorosis.

## Data Availability

The data that support the fndings of this study are available from the corresponding author upon reasonable request.

## Abbreviation

TFI: Thylstrup and Fejerskov Index
